# A rare case of disseminated cutaneous zoster in an immunocompetent patient

**DOI:** 10.1186/1471-2296-6-50

**Published:** 2005-12-14

**Authors:** S Gupta, A Jain, C Gardiner, SK Tyring

**Affiliations:** 1 Department of Medicine, University of Texas Health Science Center at Houston, 6431 Fannin, Houston, TX, 77030, USA

## Abstract

**Background:**

Disseminated cutaneous herpes zoster in healthy persons is uncommon, though it has been described in immunocompromised patients.

**Case presentation:**

We describe a case of disseminated cutaneous herpes zoster in an elderly man with no apparent immunosuppressive condition. The patient was treated successfully with intravenous Acyclovir.

**Conclusion:**

We suggest that disseminated zoster can occur in an immunocompetent host and should be promptly recognized and treated to prevent serious complications.

## Background

Disseminated cutaneous herpes zoster has been described in persons with immunosuppression due to Human Immunodeficiency Virus (HIV), hematological malignancy, or chemotherapy. However, it is uncommon to see dissemination of zoster in healthy individuals. In this report, we describe the clinical course of a patient who presented with disseminated cutaneous zoster in the absence of a known immunosuppressive condition. A brief review of literature on this topic is also presented.

## Case Presentation

A previously healthy 69-year old man presented with a 5-day history of severe pain over the right forehead and a 3-day history of vesicular eruption over right forehead, right eyelid, and nose. It was followed by the spread of vesicular eruption to involve chest, back and bilateral upper and lower extremities over next 2-days. Review of systems was negative. The patient did not have a history of chickenpox during childhood or any recent exposure to it. There was no past history of diabetes, cardiac or pulmonary disease, or lymphoma. The patient has not been on immunosuppressive or other medications.

**Figure 1:**
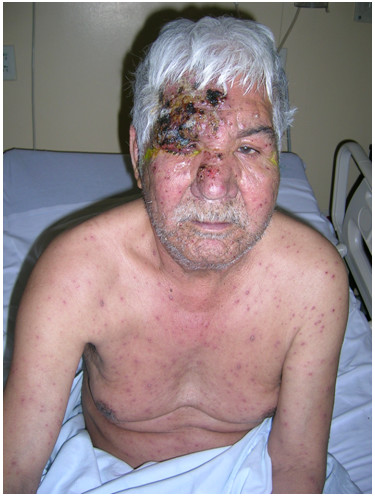
Picture of the patient showing diffuse vesicular, pustular and crusted lesions with concentration of the eruption in ophthalmic division (V1) of trigeminal nerve.

On examination, the patient was afebrile (37.4°C). He had vesicles and pustules, with crusting and swelling, in the distribution of the ophthalmic division of the trigeminal nerve (V1) including its nasociliary branch. Both eyelids were swollen, tender and red. There was no corneal involvement. Vesicles, pustules and scabs in various stages were also present over trunk and extremities (Figure 1). Palms and soles were spared and there was no lymphadenopathy. Pulmonary, cardiovascular and abdominal examinations were normal.

Complete blood count, peripheral smear, routine biochemistry, liver function tests, and chest x-ray were normal. CD4 and CD8 lymphocyte counts were 1.34 × 10^9^/L and 0.61 × 10^9^/L respectively (CD4:CD8 ratio = 2.2). Serology for Human Immunodeficiency Virus (HIV), Hepatitis A, Hepatitis B, and Hepatitis C were negative and RPR was non-reactive. The blood and vesicle culture for Varicella Zoster Virus (VZV) were negative. Serum VZV Immunoglobulin G (IgG) and Immunoglobulin M (IgM) done six days after the onset of dissemination were positive (the titers were not obtained). Skin biopsy from fresh vesicle on the trunk and forearm showed ulceration with acute inflammation, necrosis and intranuclear inclusions within epithelial cells. The direct fluorescence antigen staining from skin lesions was positive for VZV.

The patient was started on intravenous Acyclovir 800 mg every 8 hours. In the next 24 hours, the eruption of new vesicles ceased, the rash started to resolve and his clinical status improved. I.V. Acyclovir was continued for four more days and the patient discharged on oral acyclovir 800 mg 5 times daily for 16 days.

## Discussion

Herpes zoster, also called shingles is the consequence of reactivation of latent VZV from the dorsal root ganglia. It is characterized by unilateral vesicular eruptions within a dermatome. Disseminated cutaneous zoster has been defined as more than 20 vesicles outside the area of the primary and adjacent dermatomes [1]. This complication of zoster has been described in immunocompromised persons (HIV, cancer, patients on immunosuppressive therapy) and reported to be as common as 10% – 40% [1,2]. However, disseminated cutaneous zoster in otherwise healthy persons who are not on immunosuppressive therapy and have no underlying cancer is rare. In our search on PubMed using the keywords "zoster", "disseminated zoster", "immunocompetent", we could identify less than ten cases with disseminated zoster in otherwise healthy persons [3-5].

Our patient presented with characteristic skin findings of disseminated cutaneous herpes zoster. Dissemination occurred by third day of the eruption. The serology and skin biopsy findings supported the clinical diagnosis of VZV. VZV IgM is extremely rare during VZV reactivation, and even if positive, the titers are not very high. The vesicle culture for VZV was negative, however VZV is a fastidious virus and the sensitivity of culture is approximately 20% [6].

In our patient, significant age related depression in cellular immunity could have contributed to the dissemination of herpes zoster. Elderly patients should be recognized as a group in whom the risk of dissemination is higher than the average immunocompetent host. Patients with cutaneous dissemination of VZV are at risk of infection of visceral organs, particularly lungs, liver and brain. Other complications include corneal ulceration and post herpetic neuralgia[1]. Therefore, identification and aggressive treatment of disseminated herpes zoster infection in elderly immunocompetent hosts is important. The treatment of choice for disseminated zoster is intravenous Acyclovir 10 mg/kg every 8 hours for 5–7 days.

## Conclusion

Disseminated herpes zoster is a potentially serious infection that can present in the absence of immunosuppression. Early diagnosis and aggressive treatment with intravenous acyclovir can reduce morbidity and severity of complications.

## Competing interests

The author(s) declare that they have no competing interests.

## Authors' contributions

SG took care of the patient described in the report, drafted the manuscript, and edited the report. AJ did review of literature, was involved in writing the manuscript, and editing the figures. CG supervised the patient care, reviewed and edited the report. ST provided expert guidance throughout the preparation of the manuscript, reviewed and edited the report. All authors have read and approved the final manuscript.

## Pre-publication history

The pre-publication history for this paper can be accessed here:


